# *Editorial Commentary*: Melioidosis in Puerto Rico: The Iceberg Slowly Emerges

**DOI:** 10.1093/cid/ciu768

**Published:** 2014-09-30

**Authors:** David A. B. Dance

**Affiliations:** 1 Lao-Oxford-Mahosot Hospital–Wellcome Trust Research Unit, Microbiology Laboratory, Mahosot Hospital, Vientiane, Lao People's Democratic Republic; 2 Centre for Tropical Medicine and Global Health, University of Oxford, United Kingdom

**Keywords:** melioidosis, Puerto Rico, *B. pseudomallei*, Caribbean, endemic

**(See the Major Article by Doker et al on pages 243–50.)**

In this issue of *Clinical Infectious Diseases*, Doker et al describe epidemiological and environmental investigations by a joint team from the Centers for Disease Control and Prevention (CDC) and the Puerto Rico Department of Health into 2 indigenous cases of melioidosis that occurred in 2010 and 2012. The latter case had positive blood cultures and the patient survived after treatment with cefepime, which has not previously been evaluated for treatment of melioidosis; the former patient died shortly after hospital admission, presumably before cultures were taken, although the evidence of *Burkholderia pseudomallei* infection (immunohistochemistry and polymerase chain reaction on postmortem tissues) was convincing. The results of these investigations confirm irrefutably that melioidosis is endemic in Puerto Rico, with 6 human cases now described and *B. pseudomallei* detected in soil, albeit in only a single sample. The route of infection in these cases was unclear, although 1 of the patients had engaged in intravenous drug use which, along with reporting skin wounds, was 1 of 2 factors associated with seropositivity among the patients' contacts on multivariable logistic regression analysis. Interestingly, this is the first time this association has been reported since melioidosis was nicknamed “morphine-injector's septicemia” nearly 100 years ago [1]. So what are the implications of this study for local, regional, and global public health?

*Burkholderia pseudomallei* has apparently been able to persist in the environment in Puerto Rico for >30 years, as the first case occurred in 1982, but melioidosis remains very rare there, or at least is recognized only rarely. Why is that? If the incidence of melioidosis throughout Puerto Rico approached that of northeast Thailand (12.7/100 000 per year) [2], then one might expect to see approximately 440 cases every year. Is it possible that so many cases could be going undiagnosed? This seems unlikely, although there are many instances of melioidosis being missed by clinicians and microbiologists unfamiliar with its features [3]. The CDC investigation found evidence of seropositivity in 6% and 25% of contacts of the 2010 and 2012 cases, respectively, suggesting that exposure to *B. pseudomallei* might actually be quite common in some places. Serology for *B. pseudomallei* is a rather blunt instrument, however. The indirect hemagglutination assay (IHA) employed has been widely used for individual patient diagnosis, and although some culture-positive patients fail to seroconvert and background seropositivity is common among the normal population of places such as northeast Thailand, both the IHA and enzyme-linked immunosorbent assay may be better diagnostic tests for melioidosis than has previously been thought [4, 5]. The IHA, however, is unstandardized, uses a crude mix of antigens, and may reflect exposure to closely related environmental saprophytes such as *Burkholderia thailandensis*, so it may overestimate exposure to *B. pseudomallei* when used for seroepidemiological studies such as this [6, 7]. One interesting aspect is that all the cases of melioidosis so far described from Puerto Rico have occurred on the east side of the main island and all the isolates of *B. pseudomallei* have been closely related genetically. This contrasts starkly with the considerable diversity of isolates found in the environment in northeast Thailand [8]. This suggests the possibility that *B. pseudomallei* may have been relatively recently introduced to Puerto Rico and may remain relatively geographically restricted, as has been suggested elsewhere [9]. Even within the environs of the residence of the 2012 patient, Doker et al were only able to isolate *B. pseudomallei* from 1 of 20 soil samples. However, although they used a method recommended in recently published guidelines [10], which has worked well with soil from northeast Thailand [11], recent unpublished evidence suggests it may not work equally well with all soils and so this investigation may have underestimated the extent of environmental contamination with *B. pseudomallei*. It would be interesting to see the results of more extensive soil sampling throughout Puerto Rico using locally validated techniques. It is certainly possible that cases of melioidosis are currently going unrecognized elsewhere on the island.

If *B. pseudomallei* is able to survive in the environment in Puerto Rico and yet melioidosis is genuinely rare when compared with some other melioidosis-endemic areas, why is this? Possible explanations include a lower concentration of organisms in the soil, less likelihood of exposure, and greater resistance of the population to infection. First, average annual temperatures in San Juan are a few degrees lower than those in northeast Thailand, whereas the annual rainfall varies considerably across Puerto Rico, so climatic differences might play a part, as might the physical, chemical, and biological makeup of the soil. Second, only 3% of the population of Puerto Rico is involved in agricultural work, and rice farming, an important risk factor for melioidosis in Thailand, is now relatively uncommon and confined to higher altitudes. Third, fundamental genetic differences in susceptibility between the populations might exist, although diabetes mellitus, the commonest predisposing factor for melioidosis, is very common among Puerto Ricans [12]. A better understanding of the factors involved could shed important light on the global ecology and epidemiology of *B. pseudomallei*.

What of the rest of the Caribbean region? Although melioidosis is usually thought of as a disease of southeast Asia and northern Australia, there is mounting evidence that it is widely distributed throughout the Caribbean islands, Central America, and South America, albeit with only sporadic cases of infection reported in the literature. It was first described among sheep, goats, and pigs in Aruba in 1957 [13]. Subsequent reports have suggested that *B. pseudomallei* may be present in parts of Brazil, British Virgin Islands, Colombia, Costa Rica, Dominican Republic, Ecuador, El Salvador, Guadeloupe, Guatemala, Haiti, Honduras, Martinique, Mexico, Panama, Peru, Trinidad, and Venezuela as well as Puerto Rico [14–24]. It is quite likely that there could be substantial underrecognition of the disease in the less well-developed parts of the region, where laboratory support for diagnosis is minimal (eg, neighboring Hispaniola). As laboratory facilities improve and more bacteria are identified accurately by methods such as matrix-assisted laser desorption/ionization–time of flight [25], more of the melioidosis iceberg may emerge. In the meantime, laboratories in potentially melioidosis-endemic areas should screen all oxidase-positive gram-negative bacilli that are not obviously *Pseudomonas aeruginosa* and that are isolated from normally sterile samples (such as blood cultures), or in heavy pure growth from other samples, to exclude *B. pseudomallei.* Latex agglutination is widely used in Southeast Asia and has high sensitivity and specificity [26], and a new lateral flow assay looks promising [27].

In recent years, melioidosis has largely garnered attention in the United States as a potential bioweapon. Naturally occurring melioidosis, however, is a genuine problem of the rural poor in some parts of the tropics, whereas the deliberate release of *B. pseudomallei* remains a theoretical, and relatively unlikely, possibility. It is clear that there are some natural reservoirs of *B. pseudomallei* on America's doorstep, although the size of the problem remains unclear and deserves further study so that susceptible individuals such as those with diabetes can be given appropriate advice about the avoidance of infection [28]. Physicians should consider the diagnosis in anyone who has spent time in the Caribbean who presents with sepsis, severe pneumonia, or abscesses, particularly if they have predisposing factors such as diabetes. Public health authorities confronted with a single case of melioidosis in someone from the Caribbean should maintain a sense of proportion and realize that they are far more likely to have contracted the infection naturally than been the victim of bioterrorism. Laboratory workers who have handled the organism before its identity is recognized should be reassured that, despite its fearsome reputation, laboratory-acquired melioidosis is extremely rare and antibiotic prophylaxis, which has never been shown to be effective anyway, should only be used judiciously following consensus guidelines [29].
